# A new framework for X-ray absorption spectroscopy data analysis based on machine learning: *XASDAML*

**DOI:** 10.1107/S1600577525005351

**Published:** 2025-07-21

**Authors:** Xue Han, Haodong Yao, Fei Zhan, Xueqi Song, Junfang Zhao, Haifeng Zhao

**Affiliations:** a Multi-disciplinary Research Division, Institute of High Energy Physics Chinese Academy of Sciences Beijing100049 People’s Republic of China; b School of Science China University of Geosciences Beijing100083 People’s Republic of China; c University of Chinese Academy of Sciences Beijing100049 People’s Republic of China; University of Essex, United Kingdom

**Keywords:** modular machine-learning framework, X-ray absorption spectroscopy (XAS), integrated data processing

## Abstract

X-ray absorption spectroscopy (XAS) is a critical analytical technique for comprehensively characterizing the electronic configurations and atomic structures of materials. The rapid growth in both data volume and complexity, driven by modern synchrotron radiation facilities, necessitates computational frameworks capable of efficiently processing large-scale XAS datasets. To address this need, we introduce *XASDAML*, a machine-learning-based platform that integrates the entire data processing workflow. The framework coordinates key operational processes, including spectral–structural descriptor generation, predictive modeling and performance validation, while facilitating statistical analyses through principal component decomposition and clustering algorithms to uncover latent patterns within datasets. Designed with modular architecture, the system enables independent modification or enhancement of individual components, ensuring flexibility to meet evolving analytical demands. Implemented through a *Jupyter Notebook*-based interface, the platform ensures accessibility for researchers. The framework is validated with two case studies: (i) copper-foil EXAFS data show that it can predict coordination numbers and radial distribution functions; and (ii) XANES spectra of the spin-crossover complex Fe(phen)_3_ uncover bond-length changes between the low-spin and high-spin states. Comprehensive validation highlights robust toolkit functionalities, including statistical descriptor analyses, spectral visualization, and prediction of widely employed structural descriptors closely reflecting local atomic environments. By establishing standardized and extensible procedures for integrating machine learning into XAS analysis, *XASDAML* enhances research efficiency, promotes richer data insights, and provides a versatile computational resource tailored to the expanding needs of XAS research.

## Abstract 2

We introduce *XASDAML*, an open-source machine-learning framework that integrates the complete X-ray absorption spectroscopy analytical workflow, enabling efficient extraction of spectral and structural descriptors and rapid prediction of structural parameters. Demonstrated through a copper system, the framework significantly streamlines X-ray absorption spectroscopy data analysis and enhances model accuracy and interpretability, facilitating deeper insight into materials characterization.

## 1. Introduction

X-ray absorption spectroscopy (XAS) has long been a fundamental technique available at synchrotron radiation facilities, enabling characterization of electronic and geometric structural properties in materials. It delivers critical insights into the absorber’s local geometric structure, oxidation states, coordination symmetry and bond distances (Rehr & Albers, 2000 ▸). These atomic-level details are essential for understanding a wide range of material behaviors, including catalytic activity (Lee & An, 2018 ▸), structural properties (Fang *et al.*, 2022 ▸) and phase transitions (Tuerxun *et al.*, 2021 ▸). To support XAS data interpretation, computational frameworks like *ORCA* (density functional theory-based quantum chemistry suite) (Neese, 2012 ▸), *FEFF* (real-space multiple-scattering simulator) (Rehr *et al.*, 2010 ▸), *MXAN* [full multiple-scattering theory analyzer for extended X-ray absorption fine structure (EXAFS) and X-ray absorption near-edge structure (XANES) data] (Benfatto *et al.*, 2021 ▸) and *FDMNES* (finite difference method near-edge structure code utilizing density functional theory) (Bunău & Joly, 2009 ▸) have been developed to simulate or refine spectral–structural correlations. Complementary approaches have also emerged for processing time-resolved XAS datasets through modified computational workflows (Zhan *et al.*, 2017 ▸).

With the development and operation of fourth generation synchrotron radiation facilities based on diffraction-limited storage rings, application of space-resolved XAS measurement with nano-probe technology and time-dependent XAS with quick scans promote the volume of XAS datasets to a level that traditional data analysis methods struggle to process efficiently. Concurrently, the growing requirement for real-time data processing at beamlines to extract structural insights and guide subsequent experiments necessitates tools capable of rapid, accurate handling of large datasets. This challenge has driven the integration of machine learning (ML) and artificial intelligence (AI) into XAS analysis, particularly enhancing automated batch processing, feature discrimination and accessibility for non-specialists. A pivotal advancement emerged from Timoshenko *et al.*, who pioneered supervised ML for refining 3D geometries of metal catalysts using XANES spectroscopy, enabling the tracking of heterogeneous catalyst structures under operando conditions (Timoshenko *et al.*, 2017 ▸). Subsequent developments demonstrate ML’s capacity to improve both accuracy and scalability in XAS interpretation. For instance, Timoshenko *et al.* (2023 ▸) employed principal component analysis (PCA) with neural networks to interpret EXAFS data, revealing how these tools can be used to enhance the interpretation of structural information and uncover new insights into material behavior. ML has also been instrumental in linking spectral features to atomic-scale parameters. Guda *et al.* (Guda *et al.*, 2020 ▸; Guda *et al.*, 2021 ▸) systematically correlated spectral descriptors (*e.g.* edge position, intensity variations) with structural metrics including coordination numbers, bond lengths and oxidation states, proving ML can convert spectral data into usable structural information to predict material properties. These efforts suggest that ML models, including multi-layer perceptron (MLP), support vector machines, convolutional neural networks (CNN) and random forests (RF), can help reveal complex structure–property relationships in challenging XAS datasets (Guda *et al.*, 2020 ▸; Kotobi *et al.*, 2023 ▸; Rankine *et al.*, 2020 ▸; Gleason *et al.*, 2023 ▸; Zheng *et al.*, 2020 ▸). Such methodologies not only deepen atomic-level material understanding but also address critical needs for high-throughput analysis and automated interpretation in large-scale XAS studies.

With the growing interest in integrating ML into XAS data analysis, several software tools combining XAS and ML have been developed. Among them, *TRixs* by Torrisi *et al.* (2020 ▸), *XANESNET* by Penfold *et al.* (Rankine *et al.*, 2020 ▸; Rankine & Penfold, 2022 ▸) and *PyFitIt* (Guda *et al.*, 2021 ▸; Martini *et al.*, 2020 ▸; Martini *et al.*, 2021*a* ▸) developed by Guda *et al.* each represent significant steps toward more automated workflows. These tools collectively advance automated workflows through capabilities spanning data preprocessing, spectral fitting, neural network implementation and structure–spectrum mapping. *TRixs* employs random forests with multiscale polynomial-based descriptors to decode spectral correlations with parameters (*e.g.* Bader charge, nearest-neighbor distances) in transition metal oxides, demonstrating how ML can streamline analysis for targeted material systems. *XANESNET* establishes systematic neural network workflows for XANES interpretation, with dataset preparation currently anchored to external repositories like the Materials Project. *PyFitIt* offers versatile regression frameworks for structural predictions while maintaining an integrated architecture designed for methodological consistency. These pioneering efforts lay essential foundations for ML-enhanced XAS analysis, with future developments poised to expand material generality, improve cross-platform interoperability, and enhance the ability to address the increasing complexity of computational tasks.

In this study, we introduce *XAS Data Analysis based on Machine Learning* (*XASDAML*), an open-source framework designed to streamline the entire XAS data processing and analysis workflow. Building on existing solutions, *XASDAML* integrates all essential modules—from simulating XAS spectra using material coordinates and constructing ML models to predicting structural descriptors and evaluating model performance. This unified approach lowers the barrier for XAS researchers who may not have extensive ML expertise; with *XASDAML*, users can generate and curate their own XAS datasets that closely match their system of interest, specify the structure geometry they wish to study, select or develop an appropriate ML model, and obtain reliable structural predictions from newly measured XAS data. The platform further empowers users through its interactive visualization toolkit and statistical analytics module, facilitating systematic exploration of spectral–structural correlations. Accessible via a user-friendly *Jupyter Notebook* interface, *XASDAML* consolidates spectral and structural calculations, data visualization, ML modeling and predictive analysis into a single platform. This design not only enables users to customize parameters within each module but also lets them monitor program execution and review outputs in real time. Ultimately, *XASDAML* facilitates high-throughput automated analysis and fosters broader adoption of ML-driven XAS research.

## 2. Program description

**Figure 1:**
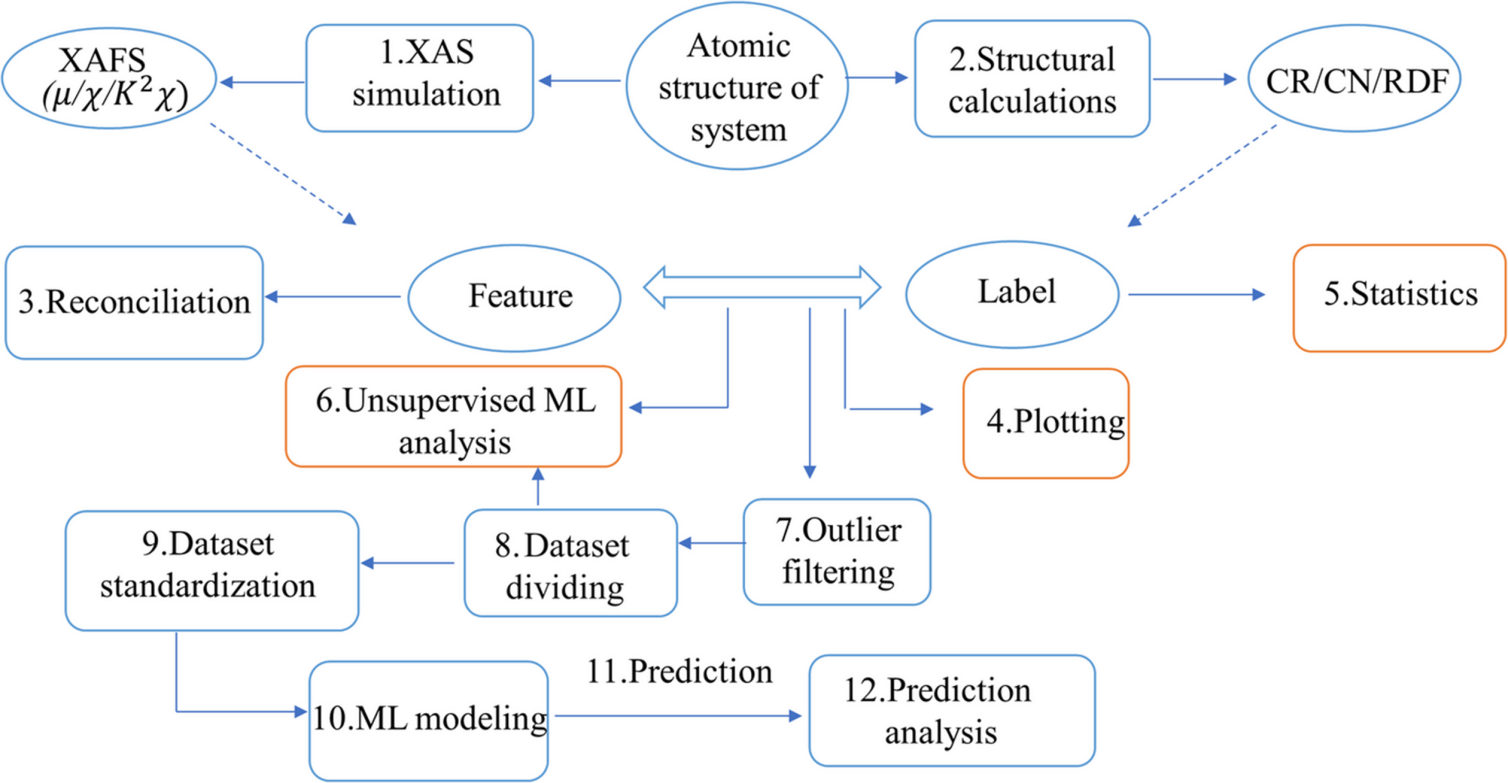
Flowchart illustrating the *XASDAML*-based workflow for XAS data analysis, from XAS calculations to model training and prediction analysis. The numbers on each module indicate the recommended execution order. Table 1 ▸ gives additional details on each module. CN refers to the coordination number of the first shell around the absorber, and CR denotes the average bond length in the absorber’s first shell; RDF refers to the radial distribution function.

*XASDAML* is an ML-based framework for XAS data analysis, consisting of 12 modules developed in Python. It is accessible through a *Jupyter Notebook* interface, offering an interactive environment that integrates code execution, data visualization and documentation for efficient, reproducible research. *XASDAML* spans the entire XAS data analysis workflow, starting with the construction of training datasets through the simulation of XAS spectra and structural descriptors from structures. These simulated data are combined into feature/label datasets, which are then divided into training, validation and test subsets after outlier filtering—facilitated by data plotting and statistical analysis. The framework then builds and trains ML models using the prepared datasets, applies these models to predict structural descriptors, and evaluates predictive performance (Fig. 1 ▸).

Most modules take the outputs of previous modules as inputs and generate files required by subsequent modules, as illustrated in Table 1 ▸. For clarity, we group the modules into four functional blocks: dataset calculation, dataset optimization, ML modeling, and prediction and prediction analysis. Supplemental modules, such as data visualization and statistical analysis, operate as plugin components to enhance core workflow capabilities. Each module maintains operational independence with five sections—introduction, library imports, parameter settings, function definitions and the main program. The parameter settings section, accessible through the *Jupyter Notebook* interface (as shown in Fig. SI1 of the supporting information), allows users to configure all necessary parameters, providing flexibility for customization and ensuring ease of use.

### 2.1. Block 1: dataset calculation (Modules 1 and 2)

*XASDAML* provides two modules for generating the features and labels required by ML models. Module 1 simulates X-ray absorption spectra, while Module 2 extracts structural descriptors, both working from the same input atomic coordinates. Although their outputs are saved by default in the same directory for convenience, each module operates independently, giving users the flexibility to run them separately or together depending on their needs.

Module 1 integrates the widely used *FEFF* package (Ankudinov *et al.*, 1998 ▸) for XAS simulations. To place experimental and theoretical spectra on a common scale, *XASDAML* employs a dedicated calibration pipeline. First, a reference sample with a well known synchrotron-determined structure is selected. By adjusting only a small set of physical parameters, we bring the calculated spectrum of this standard into close agreement with its experimental counterpart in both overall shape and peak positions. Once the optimal match is reached, the parameters are fixed and applied to all subsequent calculations, ensuring that every spectrum is generated under identical physical assumptions. Each theoretical spectrum then undergoes three successive operations. (i) Energy calibration: it is rigidly shifted so that its edge position coincides with that of the reference spectrum, thereby removing any Δ*E*_0_ offset. (ii) Interpolation to a common grid: the spectrum is resampled onto exactly the same energy mesh used for the experimental data. (iii) Edge-step normalization: intensities are scaled so that the edge jump matches the experimental scale. After calibration, interpolation and normalization, any experimental spectrum can be fed directly into *XASDAML* and compared with the theoretical database on an identical energy and intensity scale. In this way the framework delivers results that are fully comparable with those obtained by traditional methods.

After reading the input structure, the module automatically generates standard *FEFF* input files. Users can customize simulation parameters as desired, and the module, by default, calculates the standard absorption coefficient μ(*E*), the normalized EXAFS function χ(*k*), where the abscissa at χ(*E*) = [μ(*E*) − μ_0_(*E*)]/μ_0_(*E*) is converted from energy to wavenumber *k*, and the wavelet transform data *wt*. All these outputs serve as potential features for subsequent ML tasks. A progress bar and process window assist users in monitoring real-time simulation status—a valuable feature for large-scale datasets. Moreover, the module offers parallel processing options that can be tailored to the user’s computational resources. Compared with other simulation tools (Filipponi & Cicco, 2000 ▸), it is suitable for high-throughput calculation requirements. The module is not restricted to *FEFF*-generated data: users may alternatively run *FDMNES* calculations and feed the resulting μ(*E*), χ(*k*) and related files into the same directory. As long as the file names follow the expected convention, the downstream interpolation, filtering and ML modules, allowing *FDMNES* outputs to slot seamlessly into the *XASDAML* workflow.

Module 2 calculates structural descriptors that are commonly analyzed in XAS studies, including CN, CR and RDF. While CN and CR serve as fundamental parameters in EXAFS refinement, RDF offers atomistic-level insights into spatial atomic arrangements. Additional descriptors such as geometric symmetry can be integrated with minimal effort, as each descriptor is calculated independently. The module integrates six algorithms—effective coordination number algorithm (EconNN), O’Keeffe’s Voronoi coordination method (VoronoiNN), crystal nearest neighbor method (CrystalNN), minimum distance algorithm (MinimumDistanceNN), Jmol algorithm (JmolNN), and Brunner method (BrunnerNN) (Pan *et al.*, 2021 ▸)—which enable researchers to identify neighboring atoms through material-specific method selection. As with Module 1, computations preserve original sample indexing, incorporate real-time progress tracking and support parallel processing acceleration.

### 2.2. Block 2: dataset optimization (Modules 3, 7, 8 and 9)

Block 2 refines the spectra and structural descriptors produced in Block 1, ensuring that the data are of sufficient quality and consistency for training ML models in Block 3. This block reconciles simulated data (Module 3), filters outliers (Module 7), divides the dataset into training, validation and test subsets (Module 8), and standardizes the data (Module 9). Together, these steps promote robust and flexible dataset management while enhancing overall model performance. Module 3 interpolates spectra containing inconsistent energy points to preserve data smoothness and integrity. It also offers plotting tools that enable users to visually inspect their results and manually exclude any anomalous data. For a more direct overview of potential outliers, Module 4, discussed later, provides statistical summaries of all descriptors, making it easier to identify and isolate problematic samples. Module 7 applies user-defined filters, such as thresholds on CN or CR, to eliminate unphysical data. For instance, if certain algorithms (*e.g.* JmolNN) occasionally assign a CN of 15 to copper atoms—an improbable value for well ordered Cu clusters—those samples can be flagged and removed. The program preserves the original dataset and logs the indices of filtered samples in a separate file, copying the excluded spectra and structural descriptors to a distinct directory. This approach not only streamlines iterative data exploration—allowing quick adjustments to filter parameters and reanalysis—but also retains full traceability of the original data.

Following data cleanup, Module 8 partitions the dataset into training, validation and test sets according to user specifications (defaulting to a 7:2:1 ratio). To ensure efficient I/O, both the spectra and structural descriptors are saved into a single file. Module 9 then standardizes these subsets, handling data normalization and optional PCA transformations to remove discrepancies in feature scales. Key parameters (*e.g.* mean, standard deviation) are stored to maintain consistency in future predictions, making it straightforward to apply the same transformations to new or updated data.

### 2.3. Block 3: ML model (Module 10)

Block 3 implements supervised ML, providing a framework for training models, optimizing hyperparameters and saving the best-performing model for prediction. This is a time-consuming stage in the *XASDAML* pipeline, second only to the spectral simulations performed in Module 1. The computational cost is largely determined by the chosen ML model, the model architecture and the criteria for convergence. *XASDAML* offers three widely used ML model types for XAS data analysis: MLP, CNN and RF. Each model provides configurable hyperparameters, including the number of layers and nodes for MLP, the convolution kernel size and the number of channels for CNN, and the number of trees and tree depth for RF. These predefined configurations balance accessibility for non-experts with sufficient flexibility to adapt architectures to specific datasets and experimental objectives. To prevent overfitting and improve generalization, both MLP and CNN models support L2 regularization. The early stopping mechanism is integrated into the training process to save computational resources. This feature halts training when model performance no longer improves over several epochs, as monitored by the validation set’s loss value. Additionally, model checkpointing ensures that the model parameters are saved at the optimal performance point, so the final model produced is the one with the best predictive accuracy. The module also provides built-in tools to track and visualize the training dynamics, including loss curves and key performance metrics like mean squared error (MSE) and mean absolute error (MAE). These visual analytics tools enable rapid identification of underfitting/overfitting patterns through divergence monitoring between training and validation curves. For beginners, the module is designed to streamline the workflow, while offering sufficient flexibility for advanced users to customize their models for specific XAS data analysis needs.

### 2.4. Block 4: prediction and prediction analysis (Modules 11 and 12)

Block 4 focuses on the model prediction, where the trained ML models are applied to predict structure descriptors from measured XAS spectra and subsequently evaluated for their performance. This stage operates through two sequentially linked modules: the prediction module (Module 11) processes spectral inputs to generate structural predictions, while the prediction analysis module (Module 12) systematically evaluates model performance and provides interpretative insights. Module 11 imports a well trained ML model and uses it to predict structure descriptors based on input spectra. The module supports both single and batch input formats. It processes the spectra and outputs the predicted structure descriptors, but it does not assess the quality of these predictions. This separation allows users to maintain a clear workflow separation between prediction generation and subsequent quality evaluation.

Module 12, on the other hand, takes the output from Module 11 and evaluates the performance of the trained ML model. It compares the predicted values with the corresponding ground truth (if available) and computes a range of common statistical indicators to assess model accuracy. Key evaluation metrics such as MSE, MAE, determination coefficient (*R*^2^), accuracy, recall, F1 score, precision and area under the curve (Debray *et al.*, 2017 ▸; Vihinen, 2012 ▸) are available for users to choose according to the pattern of model (regression or classification) and the data pattern. Analytical outputs combine tabular statistical summaries with visual comparisons including parity plots and residual histograms, enabling rapid identification of systematic prediction errors. The module’s model comparison feature further allows side-by-side performance benchmarking across multiple architectures using shared validation datasets. Additionally, Module 12 includes an optional feature for unsupervised ML analysis, providing *K*-means clustering and PCA to further explore the relationship between the input spectra and the model’s prediction accuracy. *K*-means clustering categorizes spectra based on prediction residual magnitudes, revealing structural domains where models exhibit enhanced or diminished accuracy. Similarly, PCA provides a way to visualize the spectra in terms of their principal components, with a scatter plot colored according to the performance of the corresponding structure descriptors. These dual approaches provide mechanistic insights into model decision patterns, particularly valuable when ground truth references are limited.

In summary, Block 4 delivers both quantitative performance assessment and qualitative interpretation tools. By coupling traditional statistical validation with dimensionality-reduced analysis, it addresses critical needs in XAS computational workflows—verifying model trustworthiness while illuminating the physical basis of ML predictions in materials characterization applications.

## 3. Toolkits (Modules 4, 5 and 6)

*XASDAML* incorporates supplementary visualization and analytical toolkits that extend beyond core computational workflows, significantly enhance the understanding of datasets. These modules are designed to facilitate the inspection, interpretation and management of the data, thus supporting more informed decision making in the analysis of XAS data.

### 3.1. Toolkit 1: data visualization (Module 4)

Module 4 provides a dedicated toolkit for visualizing spectra, wavelet data and structure descriptors. This visualization is crucial for gaining an intuitive understanding of the data’s key features, helping users to identify patterns, detect anomalies and decide which datasets should be flagged as unphysical in the data filtering process. The module supports the visualization of a variety of datasets, including μ spectra, χ spectra, Fourier transform/reverse Fourier transform of χ, wavelet data and RDF for structural descriptors. Given the large volume of data, especially when working with wavelets, users can limit the range of datasets to be plotted by specifying start and end indices, as well as the interval (*e.g.* plotting every *n*th dataset). This option minimizes the number of plots and ensures that users can still capture the essential features of the data.

Additionally, Module 4 provides the ability to generate scatter plots comparing CN and CR from different methods within the same figure, allowing users to visually compare simulation performance across algorithms. Since the module runs within a *Jupyter Notebook* environment, users can easily modify and adapt the code to generate custom plots tailored to their needs. All outputs are automatically saved in time-stamped folders, ensuring that results are organized and easily retrievable.

### 3.2. Toolkit 2: statistics for structure descriptors (Module 5)

Module 5 delivers robust statistical characterization of one-dimensional descriptor datasets while preserving data integrity. It generates fundamental distribution metrics—including population counts, extremal values, central tendencies (mean, median) and dispersion measures (variance)—presented through structured tabular summaries for rapid dataset profiling. The module also supports various visualization techniques to represent the data distribution. Histograms provide insight into the frequency distribution, helping users assess central tendencies, spread and potential outliers. Density distribution graphs offer a smoothed representation of the data, allowing for the identification of underlying trends. Box plots summarize key statistics such as the median, quartiles and outliers, offering a compact yet powerful tool for comparing the variability of different datasets.

Module 5 not only provides statistical summaries but also enables users to visualize the data both before and after outlier filtering (Module 7) and dataset partitioning (Module 8). This functionality facilitates direct comparisons of how these processes influence the dataset. For continuous data, it is advisable to set a specific count step in the ‘parameter set zone’ (default is 10) to avoid arbitrary grouping, ensuring more meaningful visualizations. As with other modules, all outputs are automatically saved in time-stamped folders, allowing for efficient result management. Overall, this module delivers a comprehensive yet flexible approach to understanding the data’s statistical characteristics without modifying the original datasets.

### 3.3. Toolkit 3: unsupervised ML analysis for spectra (Module 6)

Module 6 in *XASDAML* integrates two unsupervised ML techniques—*K*-means clustering and principal component analysis (PCA)—to assist in analyzing X-ray absorption spectra. Its primary function is to visualize data distributions, especially in two-dimensional spaces. By offering both clustering and dimensionality reduction, this module uncovers hidden patterns in the data, identifies outliers and simplifies complex datasets for further analysis.

*K*-means efficiently groups spectral data based on similarity. In Module 6, the elbow method is used to determine the optimal number of clusters by plotting the intra-cluster sum of squared errors (SSE) across varying cluster counts, helping researchers pinpoint where additional clusters provide diminishing returns (Nainggolan *et al.*, 2019 ▸). Once the optimal cluster count is selected, the toolkit assigns labels to each sample, saves these labels into a new CSV file, and visualizes each cluster’s spectra in separate graphs. This approach helps researchers identify outliers and understand how different groups of data may represent distinct physical or chemical states of the material (Kartashov *et al.*, 2021 ▸).

PCA is a technique used for dimensionality reduction, enabling the projection of complex, high-dimensional data into a lower-dimensional space. By retaining as much of the original variance as possible, PCA helps simplify data interpretation while preserving essential information. Module 6 visualizes the first two principal components, which often explain over 80% of the dataset’s variance, and displays the explained variance ratio to show how much information each principal component retains (Kartashov *et al.*, 2021 ▸; Andrejevic *et al.*, 2022 ▸) (as introduced in the supporting information). Scatter plots map each sample to a point, enabling easy color-coding based on clustering groups or structural descriptors. This approach highlights how well different spectra and structural features align in the reduced dimensional space.

When combining PCA with *K*-means clustering, users can observe group structures and identify trends or anomalies that may not be apparent in the higher-dimensional space. The toolkit not only analyzes spectral distributions but also the relationship between the spectra and corresponding structural descriptors (such as CN and CR). After clustering the spectra with *K*-means, the toolkit uses box plots to display the distribution of structural data for each cluster, making it easy to compare the structural characteristics within and between clusters. This vertical comparison helps researchers explore how different groups of spectra are associated with different structural features. Moreover, after performing PCA, Module 6 enables comparisons between spectra and their corresponding structural descriptors. By plotting the first two principal components in a scatter plot, users can observe how different spectral patterns (*e.g.* μ and χ spectra) relate to variations in structure. The color coding of the scatter plot can reflect the clustering results or structural data, providing insights into how well the first two principal components explain the relationship between spectra and structure. For example, when using *K*-means clustering on the scatter plot, researchers can visually inspect the data for significant groupings or abnormal data points, aiding in the interpretation of complex spectral data and the identification of outliers.

*K*-means clustering and PCA are both widely used in data processing and analysis. PCA, for instance, is commonly employed to reduce dimensionality, extract key features for supervised ML, and simplify spectral datasets. Clustering with *K*-means helps reveal differences among data groups, facilitating outlier detection and offering insights into a material’s chemical state (Kartashov *et al.*, 2021 ▸). When combined with relevant physical information, these methods support effective data preprocessing and elucidate the physical properties of XAS spectra (Andrejevic *et al.*, 2022 ▸).

The visualizations produced by PCA and *K*-means clustering in Module 6 also aid in feature selection for ML. Observing how principal components and clusters align with structural descriptors reveals which features may be most important for training predictive models (Mishra *et al.*, 2017 ▸). Similar to Module 5, Module 6 allows users to analyze datasets at different stages of preprocessing, such as before and after outlier screening (via Module 7) and data partitioning (via Module 8). This flexibility enables users to compare how the spectral and structural distributions evolve after these steps, facilitating the refinement of data before building predictive models. The toolkit can process both CSV datasets (prior to partitioning) and TXT datasets (after partitioning) to ensure that users can perform analysis on datasets at various stages, including the training, validation and test sets.

In summary, Module 6 in *XASDAML* offers a comprehensive set of tools for unsupervised analysis of X-ray absorption spectra. By integrating *K*-means clustering and PCA, this module allows users to explore the distribution of spectra, investigate relationships between spectra and structural descriptors, and gain valuable insights for model building. The visualization of clustering and principal components aids in understanding complex data structures, identifying outliers and selecting relevant features for subsequent ML tasks. These capabilities enhance the overall workflow, helping researchers better interpret their data and improve the performance of ML models.

## 4. Application

To showcase the capabilities of *XASDAML* we first consider a copper system. Copper is a widely used metal in electrochemical materials; its foil is in the indispensable standards at the XAS beamlines that is used to calibrate the absorption edge of XAS in the measurement. The structural database in this work was generated by classical molecular-dynamics (MD) simulations performed with the *GULP* code (Gale & Rohl, 2003 ▸) and the Mishin embedded-atom potential. A 108-atom Cu cluster was energy-minimized, gradually heated to sample disordered configurations, and then cooled back to room temperature. Atomic coordinates were saved at regular intervals during the MD run, yielding 5000 snapshots (Fig. SI2); these snapshots were subsequently fed into *FEFF* to build the Cu EXAFS data set. Simulation parameters were set with reference to the experimental Cu-foil spectrum: EDGE K (Cu *K* edge); EXAFS 20 Å to include long single-scattering paths; RPATH 7 Å together with NLEGS 8 to retain multiple-scattering trajectories of up to eight legs within a 7 Å cluster radius; a self-consistent potential sphere of SCF 6.8 Å 0 30 0.1 1 (6.8 Å radius); exchange–correlation set to EXCHANGE 0 0 0.6 (Hedin-Lundqvist potential plus a 0.6 eV imaginary broadening); and Debye–Waller damping defined by DEBYE 343 K 300 K.

Fig. SI3(*a*) illustrates the calculation of absorption spectra and feature extraction. Multiple representations of the spectra are calculated, including the absorption spectrum μ(*E*), the fine-structure χ(*k*) spectrum, its *k*^2^-weighted form [*k*^2^χ(*k*)], the Fourier transform into real-space χ(*R*), and the wavelet transform (WT) representation. Fig. SI3(*b*) illustrates how *XASDAML* organizes and stores these data. Fig. SI3(*c*) shows an example of the automatically extracted spectral features. On the structural side we focus on local-environment descriptors—CN, CR and RDF. To ensure robustness and generality we evaluated all neighbor-finding algorithms bundled with *XASDAML* (EconNN, VoronoiNN, JmolNN, MinimumDistanceNN, CrystalNN and BrunnerNN_relative). Fig. SI3(*d*) compares descriptor values returned by the different algorithms, while Fig. SI3(*e*) presents CN and CR distributions from JmolNN and CrystalNN. The *XASDAML* platform provides essential statistical analyses and visualization toolkits that support us in understanding structural descriptors, such as CN, CR and RDF. A clear statistical understanding of these structural descriptors increases confidence in the rationality of simulated spectra and subsequent analyses. After obtaining the raw datasets, a series of statistical metrics—such as sample size, mean, median, quartiles, variance and range—are computed, complemented by automated visualizations including bar plots, density distributions and box plots. Fig. SI4 illustrates how the CN and CR distributions vary with the choice of neighbor-finding algorithm, while the accompanying tables provide the numerical detail. Table SI1 summarizes the CR delivered by each algorithm, and Table SI2 lists the corresponding CN statistics. Together, these graphical and tabular outputs allow us to assess data quality at a glance, pinpointing anomalous structures or outliers.

**Figure 2:**
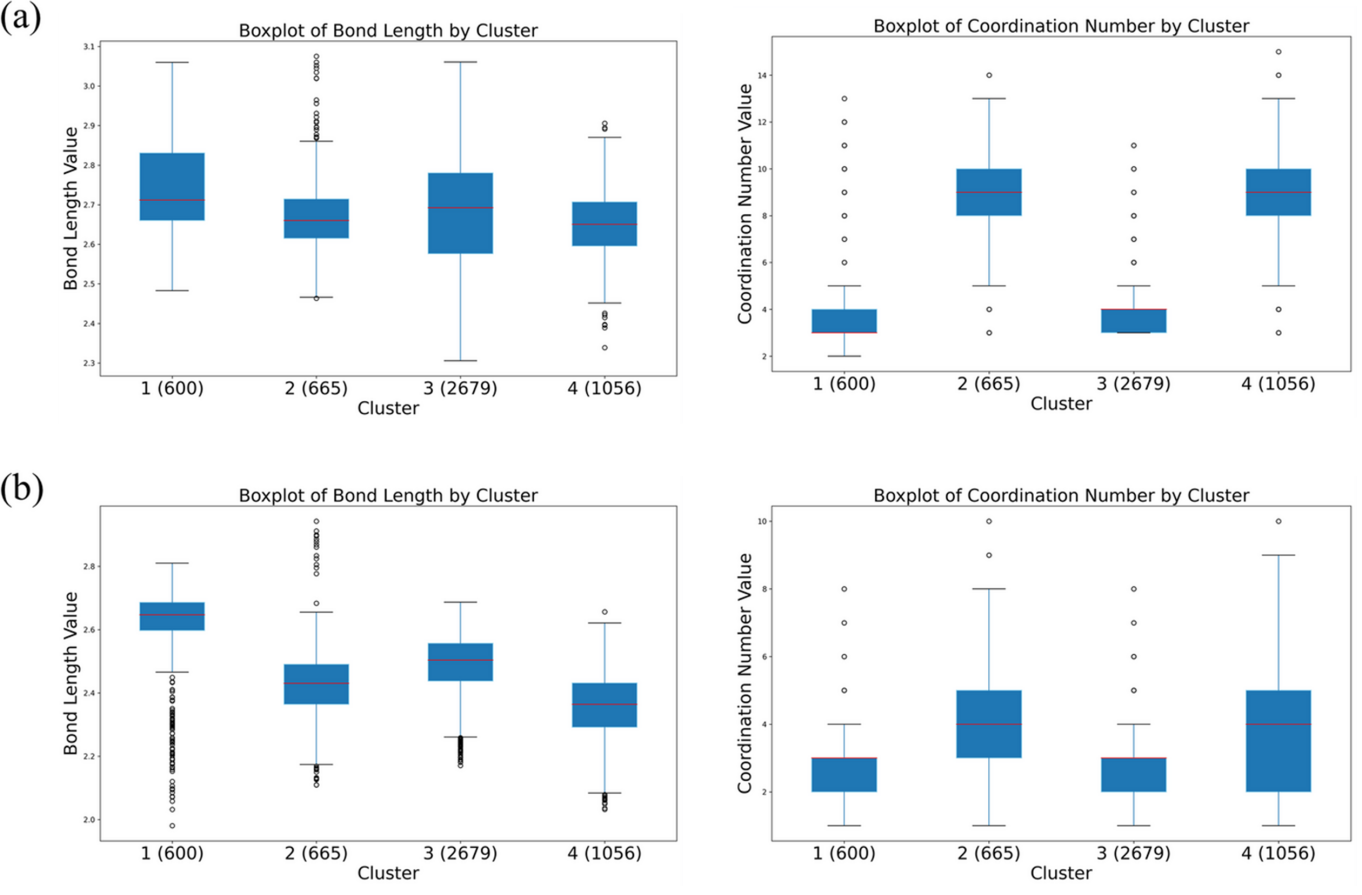
Box plots illustrating the distribution of CN and CR, calculated using (*a*) JmolNN and (*b*) MinimumDistanceNN methods, within the four *K*-means clusters of μ spectra.

Understanding how spectral fingerprints relate to structural descriptors helps to uncover the intrinsic patterns hidden in complex XAS datasets. Using the μ spectra as a demonstration, we first performed PCA for dimensionality reduction and plotted the first two principal components; together they already account for more than 70% of the total variance (Fig. SI5*a*). We then applied *K*-means clustering to the μ spectra. As an initial pass the data were partitioned into three clusters, and the corresponding spectra were overlaid [Figs. 2 ▸(*a*) and 2(*b*)] to flag any potentially anomalous lineshapes. Guided by the elbow method, the number of clusters was subsequently set to four; the μ spectra in each group were examined in detail and box-plots of CN and CR were drawn for every cluster (Fig. 2 ▸). The results show that the JmolNN neighbor-finding scheme matches the structural characteristics of this system most closely, and that the μ spectra are markedly more sensitive to CN than to CR. To visualize how the first two PCs correlate with CN and CR, we colored the PC1–PC2 scatter plot by the first-, second- and third-shell CN or CR values (Fig. SI6). Once again the μ spectra proved highly sensitive to CN: when colored by CN the points are clearly separated, whereas coloring by CR gives a much weaker discrimination—confirming that μ(*E*) captures CN-related structural variations far more effectively than those associated with average bond length.

After this preliminary mapping between spectral features and structural descriptors, we applied the filtering module to remove unrealistic samples and improve data quality. Thresholds of the first shell were set to CN = 2–12 and CR = 2.4–3.0 Å; any structures outside these windows were discarded. The remaining spectra and descriptor data were split into training, validation and test subsets in a 7:2:1 ratio and saved in TXT format for subsequent modeling.

**Figure 3:**
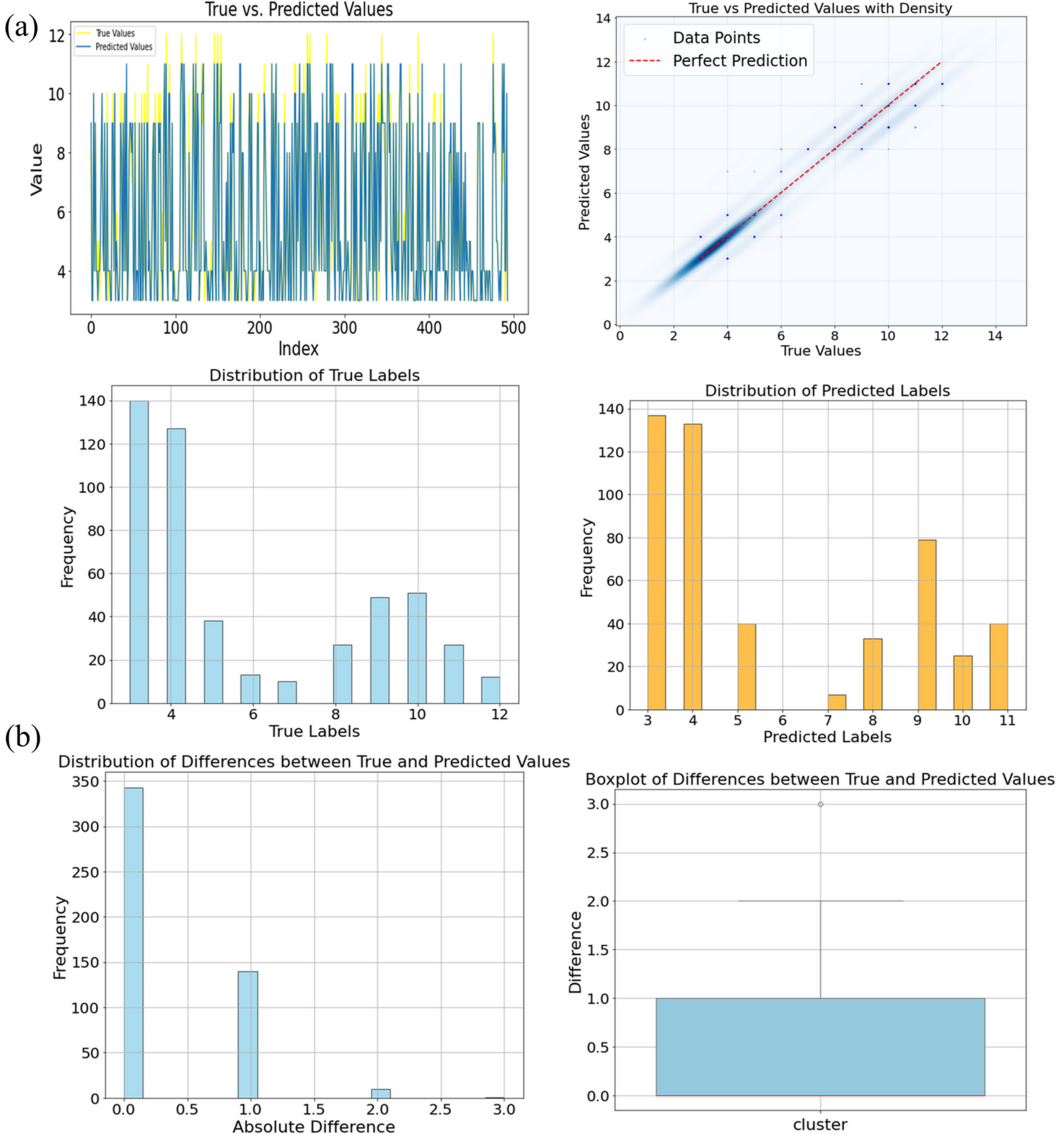
Prediction result for the Cu system using *XASDAML*. (*a*) Bar chart comparing predicted and true CN values, alongside parity plots and line charts, illustrating the model’s predictive accuracy. (*b*) Bar chart and box plot of prediction errors.

After data partitioning and standardization, the machine-learning module offers three widely used supervised models—MLP, CNN and RF. For an initial demonstration we trained a classical MLP that takes the μ spectrum as input and predicts the CN. During training the platform automatically stores the network architecture, the training/validation loss curves and the optimized weights, ensuring full traceability for later comparison or re-use. Convergence analysis (Fig. SI7) shows that a dataset of 5000 samples is already sufficient for reliable first-shell CN learning. The resulting model achieved an *R*^2^ of 0.95 with a MSE of approximately 0.37 for the first shell (Fig. SI8); predictive accuracy decreases for the second and third shells (Fig. SI9). Fig. 3 ▸ juxtaposes the predicted and true CN values as bar, scatter and line plots, providing an intuitive view of the model’s performance. To probe the error landscape we applied *K*-means clustering and PCA to the residuals. Fig. SI10 displays the error distributions within the spectral clusters identified by *K*-means together with their representative μ traces, while the PCA score plot is color-mapped by prediction error. Points with the largest errors form clear outliers, suggesting that targeted re-sampling of these regions could further boost model accuracy. Then, we fed an experimental Cu-foil spectrum into the trained model. The network predicted a first-shell coordination number of 12, exactly matching the expected face-centred cubic value and demonstrating the model’s ability to transfer from simulated to real data.

**Figure 4:**
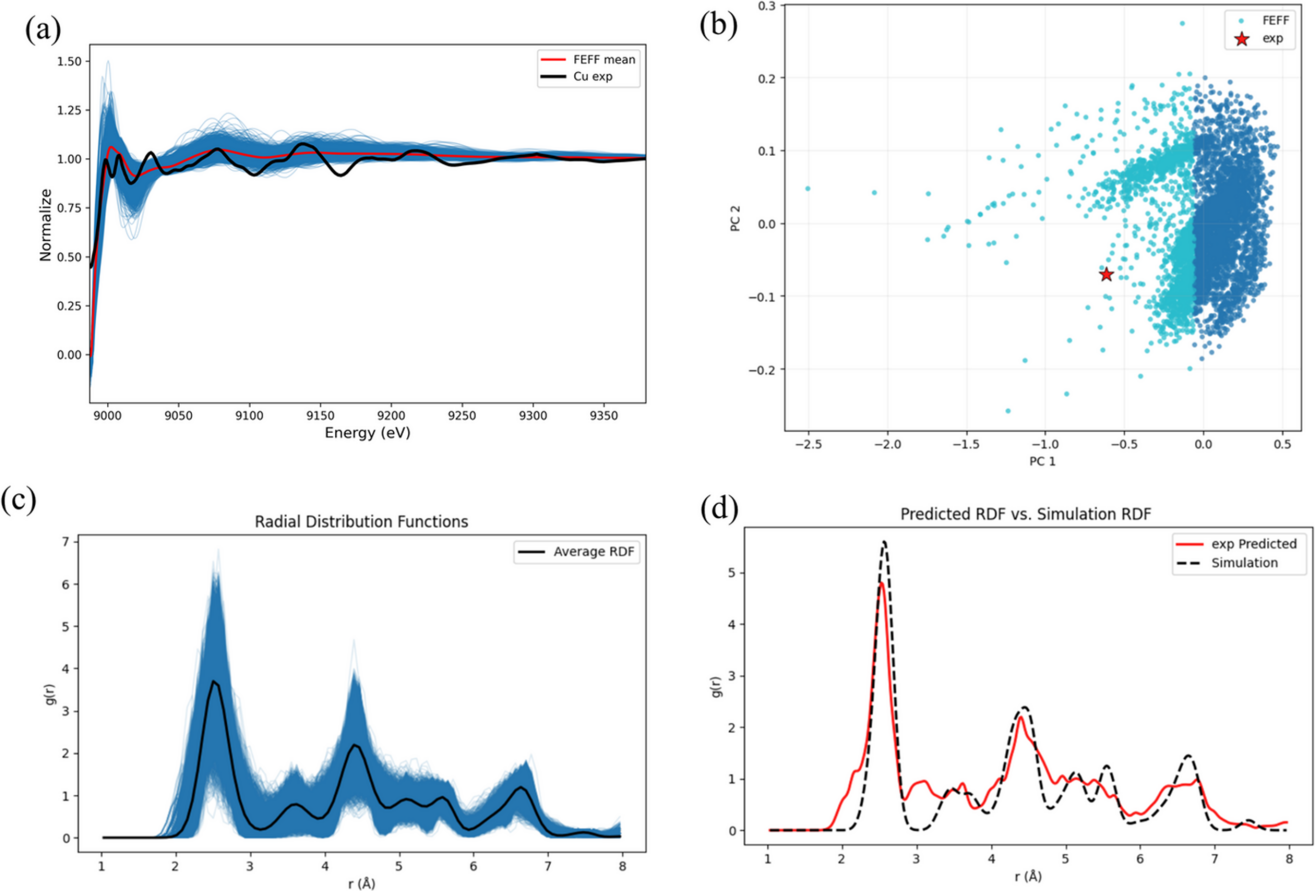
Prediction result for the Cu system using *XASDAML*. (*a*) Bar chart comparing predicted and true CN values, alongside parity plots and line charts, illustrating the model’s predictive accuracy. (*b*) Bar chart and box plot of prediction errors.

To probe how the EXAFS of thermally treated Cu relates to structural features beyond the first coordination shell, we trained a random-forest regressor that takes the normalized μ spectrum as input and predicts the RDF. Fig. 4 ▸(*a*) contrasts the full set of simulated spectra with the experimental trace, while Fig. 4 ▸(*b*) projects the experimental spectrum into the PCA space of the *FEFF* simulations, where it sits on the periphery of the spectral cloud—evidence of a moderate mismatch between calculation and experiment. The ensemble-averaged RDF of the Cu clusters is shown in Fig. 4 ▸(*c*). The model attains an MAE of 0.134. To visualize its performance, the test set was sorted by MAE, divided into eight deciles, and four curves were randomly selected from each decile; the predicted and true RDFs are compared in Fig. S11. Even in the worst decile the main peak is reproduced satisfactorily. Applying the trained model to the experimental spectrum yields the RDF plotted in Fig. 4 ▸(*d*). The first-shell peak position and coordination number match expectations; the second-shell peak is slightly shifted to lower radial distance and under-estimated in amplitude, and the third shell is likewise shifted downwards, broader and lower than theory but still recognisable. In short, despite the imperfect overlap between experimental and simulated spectral spaces, the model recovers the key bond lengths of the primary shells, with most error confined to more distant coordination shells.

We further evaluated the performance of *XASDAML* on XANES data using the spin-crossover model complex iron(II) tris-phenanthroline, FeII(phen)_3_. In its ground state, FeII(phen)_3_ is low-spin (LS); photo-excitation induces a spin-crossover to the high-spin (HS) state. This system is a benchmark in time-resolved magnetic studies and has been extensively investigated by TR-XAS. Nozawa *et al.* (2010 ▸) reconstructed the EXAFS of the photo-excited state and showed that the first-shell Fe–N bonds elongate by roughly 0.17 Å relative to the LS ground state. To provide a static HS reference, the sterically hindered analog FeII(2-CH_3_-phen)_3_ was synthesized; its methyl substituent locks the complex in an HS configuration and also serves for determining the excitation fraction.

**Figure 5:**
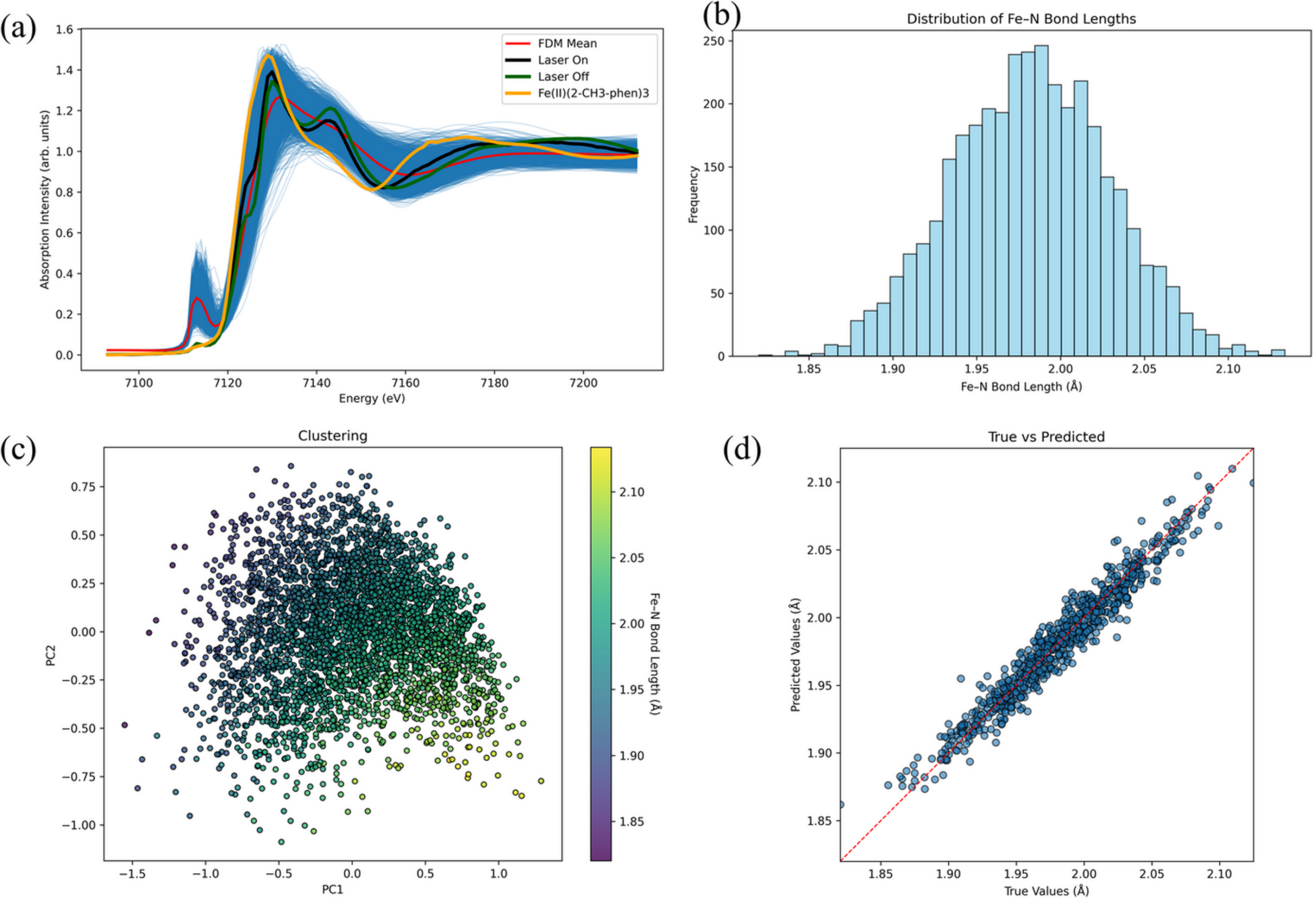
*XASDAML* workflow and ML results for Fe II(phen)_3_. (*a*) Experimental Fe *K*-edge XANES of Fe II(phen)_3_ recorded with the laser off (LS, green) and on (photo-induced HS, black), together with the static HS analog Fe II(2-CH_3_-phen)_3_ (yellow). Overlaid in blue are the *FDMNES* spectra generated from geometries in which every Fe–N bond was randomly perturbed by ±0.20 Å; the red line is their mean. (*b*) Distribution of the average Fe–N bond length obtained for the perturbed structures. (*c*) Principal-component projection (PC1 versus PC2) of all calculated spectra, color-coded by the corresponding average Fe–N bond length; each point represents one structure. (*d*) Parity plot of the ML model applied to the test set (20% of the data). The dashed line denotes *y* = *x*; the model attains *R*^2^ = 0.944 and MAE = 0.0085 Å.

TR-XAS spectra were collected at beamline 11-ID-D of the APS (see Chen & Zhang, 2013 ▸, for experimental details; Chen & Zhang, 2013 ▸). Fig. 5 ▸(*a*) displays the laser-on and laser-off spectra of FeII(phen)_3_ together with the spectrum of FeII(2-CH_3_-phen)_3_. XANES calculations were performed with *FDMNES*. The initial LS structure was taken from Yan *et al.* (2000 ▸) (see Fig. SI12*a*), and the core-hole broadening Γ_hole_ was fixed at the default iron value of 1.33 eV. The calculated spectrum agrees well with experiment (Fig. SI12*b*).

To sample a broader structural space, we randomly perturbed each Fe–N bond within ±0.20 Å around its equilibrium length, generating 3600 geometries. Their *FDMNES* spectra are plotted in Fig. 5 ▸(*a*), and the distribution of the resulting coordination numbers is summarized in Fig. 5 ▸(*b*). PCA of the computed spectra, color-coded by average Fe–N bond length, is shown in Fig. 5 ▸(*c*).

Using these data, we trained a regression model that predicts the average Fe–N bond length directly from the spectrum. On the test set the model reaches *R*^2^ = 0.944 and MAE = 0.0085 Å [Fig. 5 ▸(*d*)]. Applying the model to the experimental spectra gives an average bond length of 1.97 Å for the laser-off trace, 2.01 Å for the laser-on trace, and 2.08 Å for FeII(2-CH_3_-phen)_3_. The previously reported excitation fraction of ∼37% is reproduced (Zhan *et al.*, 2017 ▸), and the predicted bond-length trend matches experiment. The remaining discrepancy for FeII(2-CH_3_-phen)_3_ (experimental value 2.17 Å) likely arises because the training set only contained Fe(phen)_3_-type structures with altered Fe–N distances but no explicit geometries of the methyl-substituted analog. Even so, *XASDAML* captures the qualitative structural change tendency.

## 5. Discussion

*XASDAML* is built with a modular architecture that provides flexibility and adaptability in processing and analyzing XAS data. A key strength of this framework is its ability to replace or upgrade individual modules, as long as the input and output files conform to a consistent format. This design enables users to customize the system to their research needs or incorporate future advancements in XAS data analysis without disrupting the overall workflow.

Many XAS analyses benefit from enhanced feature extraction, where researchers derive more detailed descriptors from the raw absorption spectra to strengthen subsequent ML steps. Although *XASDAML* currently uses wavelet transforms as its primary method of spectral feature extraction, we have reserved a placeholder in Block 1 for a more comprehensive feature descriptor module. This planned module could incorporate additional techniques—such as polynomial fitting, the cumulative distribution function (Chen *et al.*, 2024 ▸), or intuitive descriptors of spectra (such as edge position, intensities, positions and curvatures of minima and maxima) (Guda *et al.*, 2021 ▸)—to generate richer inputs for ML models. These enhancements would offer richer inputs for ML models, deepening the analysis of spectral–structural relationships.

Graph neural networks (GNNs) have emerged as a powerful solution for modeling data that exhibit intricate relational structures, making them well suited to cases where local and interatomic interactions play critical roles. XAS data inherently involve complex interatomic relationships and local structural information, which are well suited for modeling with graph-based approaches. Preliminary applications of GNNs to XAS-related problems have shown a significant advantage in capturing intricate structural correlations (Kwon *et al.*, 2024 ▸). Notably, we have already applied GNNs to XAS-related work, and our results demonstrate their significant advantage in capturing complex structural correlations (Zhan *et al.*, 2025 ▸). Integrating GNNs into *XASDAML* would not only improve predictive performance but also broaden the framework’s applicability for materials design and discovery.

While *XASDAML* has demonstrated promising performance with simulated data, applying it to experimental datasets requires overcoming the quantitative mismatch between theoretical calculations and real measurements (Penfold *et al.*, 2024 ▸). This discrepancy arises not only because theoretical spectra depend heavily on the accuracy of computational algorithms but also due to practical issues such as instrument calibration errors, baseline intensity variations and absorption-edge shifts present in experimental data. Therefore, preprocessing steps—including normalization, standardization, spectral energy shifting and appropriate broadening—are typically required to better align simulated spectral features with experimental observations. Additionally, incorporating noise into training datasets can enhance model robustness, improving its ability to generalize effectively to experimental conditions. Given the limited availability of experimental XAS data, unsupervised domain-transfer techniques, such as cycleGANs, present a promising approach for converting simulated spectra into pseudo-experimental counterparts. These methods effectively bridge the gap between theoretical and experimental domains. If the theoretical spectra are reasonably accurate in reflecting key structural relationships, the ML approach offered by *XASDAML* can facilitate large-scale, automated parsing of spectral–structure correlations and thereby extend its utility to experimental data.

## 6. Conclusion and outlook

This study introduced *XASDAML*, a data processing framework that applies ML algorithms to streamline the analysis and prediction of XAS data. The framework integrates multiple stages, including spectral and structural calculations, data visualization, statistical analysis, model training, prediction and result interpretation. By combining ML methods with traditional XAS data processing, *XASDAML* enhances data handling efficiency and offers deeper insights into the correlation between spectral and structural properties. The modular design and open-source nature of the framework ensure broad applicability to a variety of datasets and research objectives, while the *Jupyter Notebook* interface facilitates ease of use and encourages reproducibility.

Regarding the application of *XASDAML* to Cu EXAFS, using only the μ spectrum as input, an MLP model correctly predicted the first-shell coordination number for the experimental spectrum. When the edge-step-normalized (norm) spectrum was provided instead, the model likewise reproduced the first-shell bond length and coordination number, even though the experimental and simulated data occupy somewhat different regions of feature space. Rigorous EXAFS analysis normally relies on explicitly approximating scattering amplitudes and phases (Martini *et al.*, 2021*b* ▸), or, on working directly with χ(*k*) and its wavelet fingerprints, Timoshenko *et al.* (2018 ▸) have shown that such physically motivated descriptors can enhance predictive accuracy. To keep the present Cu tutorial concise we deliberately omitted explicit amplitude-and-phase fitting, yet the simplified μ-based model still captured the essential first-shell parameters. Future releases will provide an optional feature-extraction module that automatically derives full scattering amplitudes, phases, χ(*k*) and wavelet coefficients, enabling users to choose between lightweight μ or norm descriptors and more rigorous scattering-based inputs according to their EXAFS requirements. Regarding Fe II(phen)_3_ XANES, a separate regressor trained on 3600 FDMNES simulations recovered the Fe–N bond-length elongation associated with the low-spin to high-spin crossover, achieving an *R*^2^ of 0.944 and a mean absolute error of 0.0085 Å; it therefore captured the bond-length shift tendency with high fidelity.

Compared with previous XAS-ML tools, *XASDAML* offers enhanced operational flexibility and customization by unifying all stages of data handling, thereby eliminating the need for multiple disparate tools. This streamlined approach lowers the barrier to entry for users with varying levels of expertise in integrating ML methods into the XAS data processing workflow and supports extensive customization and scalability to meet diverse research requirements. Additionally, *XASDAML* provides comprehensive features for data exploration, visualization and model selection, enabling researchers to derive more precise physical and chemical insights. Its open-source nature fosters collaboration and continuous improvement, setting it apart from earlier frameworks by offering a more integrated and adaptable solution for advanced XAS data analysis.

Despite these advancements, several challenges remain for *XASDAML*. Key areas for improvement include extracting more accurate and meaningful physical information from noisy or complex data and enhancing the framework’s generalization capabilities across different structural datasets. Moreover, strengthening the robustness of ML models through better integration of physics-based constraints, refined data preprocessing methods, improved model interpretability, and effective uncertainty quantification will be essential for reliable and accurate predictions. Future development will therefore focus on implementing advanced ML algorithms, refining interpretability of models, and optimizing data preprocessing. These enhancements will position *XASDAML* as a more reliable and effective analytical tool, capable of delivering precise, robust and efficient XAS data analysis.

## Supplementary Material

Tables S1 and S2; Figures S1 to S12. DOI: 10.1107/S1600577525005351/rv5192sup1.pdf

## Figures and Tables

**Table 1:** Modules in *XASDAML*, including their inputs and outputs The modules are grouped into four blocks—dataset calculation (Block 1), dataset optimization (Block 2), ML modeling (Block 3), and prediction and prediction analysis (Block 4)—as well as three supplementary toolkits. CN: the coordination number of the first shell; CR: the average bond length in the absorber’s first shell; RDF: the radial distribution function.

No. of module	Functions	No. of block	Input	Output
1	Simulation of XAS	Block 1	3D atomic structure of material	Spectra (μ, χ, wt)
2	Simulation of structure descriptors	Block 1	3D atomic structure of material	Structure descriptors (CR, CN, RDF)
3	Data reconciliation	Block 2	Spectra (μ)	Spectra after interpolation
4	Spectra and structure descriptors plot	Toolkit 1	Spectra (μ, χ, wt) and structure descriptors	Figures for spectra (μ, χ, wt) and structure descriptors (RDF)
5	Statistics of structure descriptor	Toolkit 2	Structure descriptors	Tables for statistical analysis and figures for structure descriptors
6	Data analysis of spectra and structure descriptors	Toolkit 3	Spectra (μ, χ, wt) and structure descriptors	Data analysis figures for spectra with structure descriptors
7	Dataset optimization	Block 2	Spectra (μ, χ, wt) and structure descriptors	Spectra, structure descriptors and index of outliers’ samples
8	Dataset division	Block 2	Spectra (μ, χ, wt) and structure descriptors and index of outliers samples	Divided dataset (training set, validation set and test set)
9	Dataset standardization	Block 2	Divided dataset (training set, validation set and test set)	Dataset after normalization or PCA transformation
10	Machine-learning modeling	Block 3	Dataset after normalization or PCA transformation	Optimal model and figure of loss curve
11	Prediction	Block 4	Optimal model and test set	Prediction of test set
12	Model performance evaluation	Block 4	Prediction, true labels, and features of test set	Statistics table of prediction value and true value and data analysis for prediction results

## Data Availability

The data and code supporting this work are available at the GitHub repository (https://github.com/BSRF-XA/XASDAML).
