# Supplementary material for: A new framework for X-ray absorption spectroscopy data analysis based on machine learning: XASDAML
Source: J Synchrotron Radiat. 2025 Jul 21;32(Pt 5):1244–56. doi: 10.1107/S1600577525005351 (PMC12416422; doi:10.1107/S1600577525005351)
Supplement: Supplementary file 1 [file s-32-01244-sup1.pdf]

JOURNAL OF SYNCHROTRON RADIATION

Volume 32 (2025)

Supporting information for article:

A new framework for X-ray absorption spectroscopy data analysis based on machine learning: XASDAML

Xue Han, Haodong Yao, Fei Zhan, Xueqi Song, Junfang Zhao and Haifeng Zhao

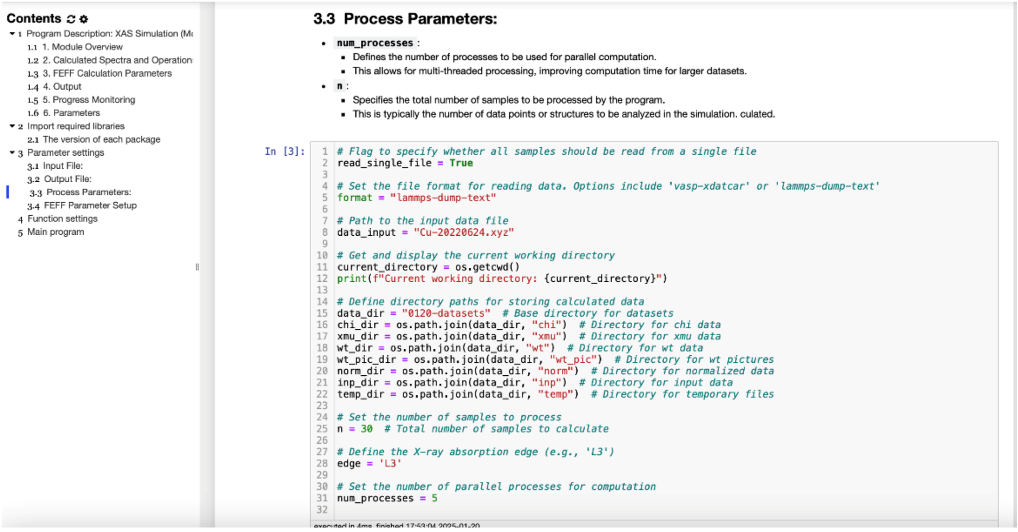

Figure S1 Screenshot of module 1(Simulation of XAS) open in jupyter notebook. On the left is the table of contents outlining the module's architecture, while the displays the code section. Here, the parameter settings section is shown, where users can configure necessary parameters and their descriptions to enable flexible analysis.

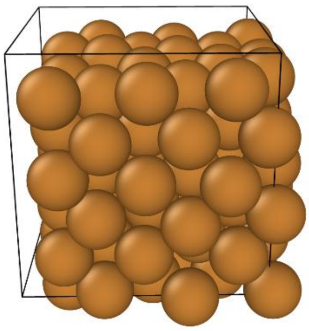

Figure S2 A representative structure from the molecular dynamics simulation of the copper system.

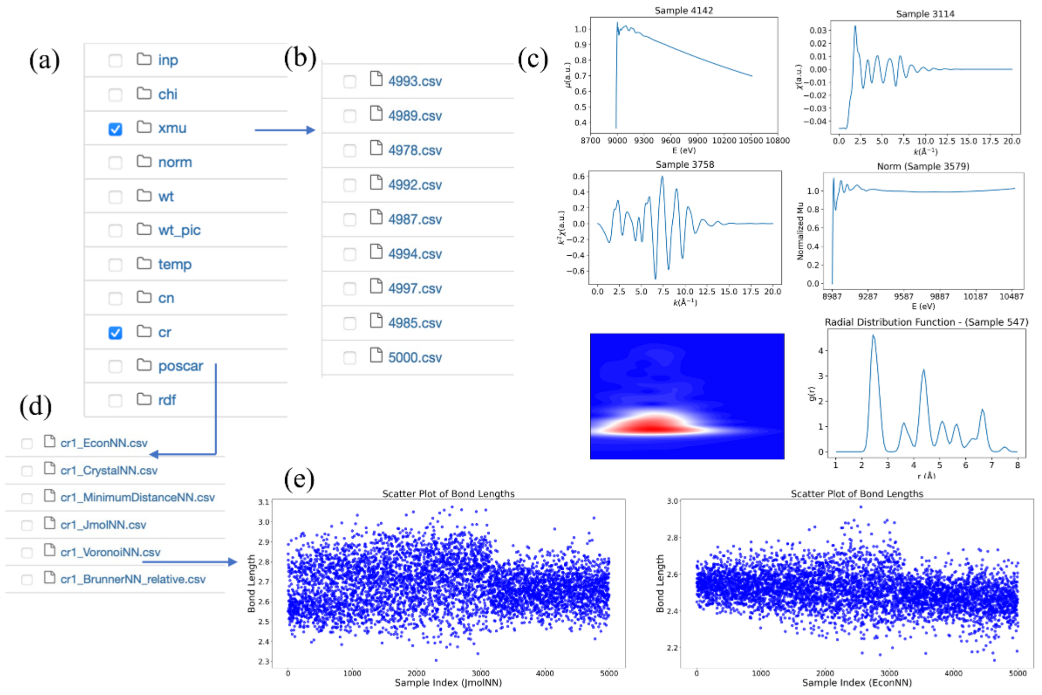

Figure S3 Dataset construction and visualization of the Cu system in XASDAML. (a) Calculation and extraction of multiple spectral features—including the absorption spectrum μ(E), fine-structure χ(k), and other related spectral forms—as well as commonly used structural descriptors(CN, CR, RDF) reflecting the local atomic environment around the absorbing atoms. (b) Screenshot illustrating the organized storage structure of output datasets within XASDAML after dataset generation and processing. (c) Visualization interface displaying extracted spectral features. (d) Screenshot of output files generated by structural descriptor calculations in XASDAML, with filenames specifying the selected neighbor-finding algorithms (EconNN, CrystalNN, JmolNN, MinimumDistanceNN, VoronoiNN, BrunnerNN_relative). (e) Distribution of calculated CR values obtained using the JmolNN and CrystalNN methods.

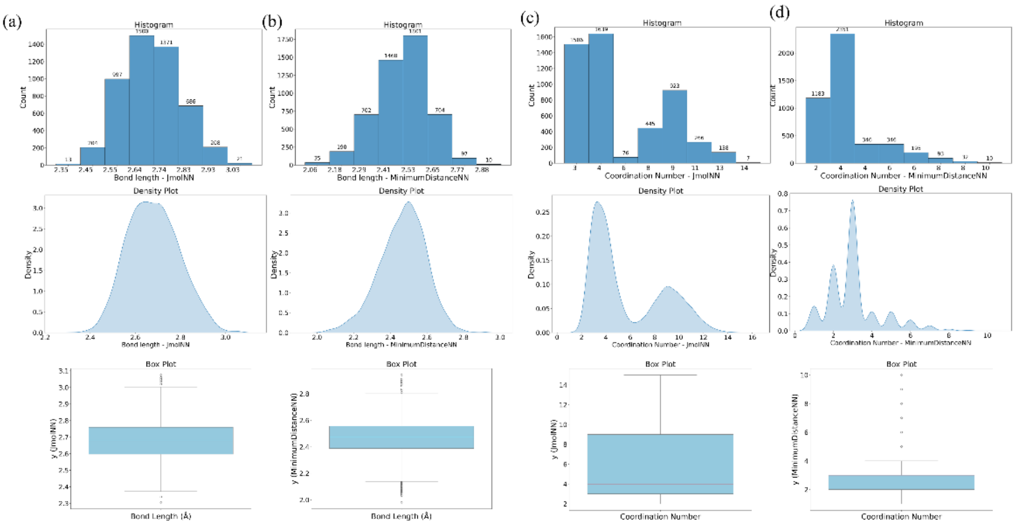

Figure S4 Histograms, box plots, and density distributions of CN and CR for the Cu system, calculated with different nearest-neighbor methods. (a) CN calculated with the JmolNN method; (b) CN calculated with MinimumDistanceNN; (c) CR obtained from JmolNN; (d) CR obtained using MinimumDistanceNN.

Table S1 Bond length statistics for different algorithms, presents the statistical properties of bond lengths calculated using various neighbor-finding algorithms.

	cr_BrunnerNN_relative	cr_CrystalNN	cr_EconNN	cr_JmolNN	cr_MinimumDistanceNN	cr_VoronoiNN
count	5000	5000	5000	5000	5000	5000
mean	2.58	2.55	2.55	2.68	2.47	6.04
std	0.13	0.1	0.09	0.12	0.13	2.37
min	2	2	2.2	2.3	2	2.4
25%	2.5	2.5	2.5	2.6	2.4	3
50%	2.6	2.6	2.6	2.7	2.5	7.5
75%	2.6	2.6	2.6	2.8	2.6	7.9
max	4.4	2.9	3.1	3.1	2.9	9.1

Table S2 Statistics of coordination numbers of different algorithms, presents the statistical properties of coordination numbers calculated using various neighbor-finding algorithms.

	cn_BrunnerNN_relative	cn_CrystalNN	cn_EconNN	cn_JmolNN	cn_MinimumDistanceNN	cn_VoronoiNN
count	5000	5000	5000	5000	5000	5000
mean	5.07	4.57	4.71	5.75	3.03	16.07
std	2.3	2.73	2.45	2.96	1.35	4.06
min	1	1	1	2	1	8
25%	3	3	3	3	2	12
50%	3	3	3	4	3	16
75%	8	7	7	9	3	19
max	37	13	12	15	10	30

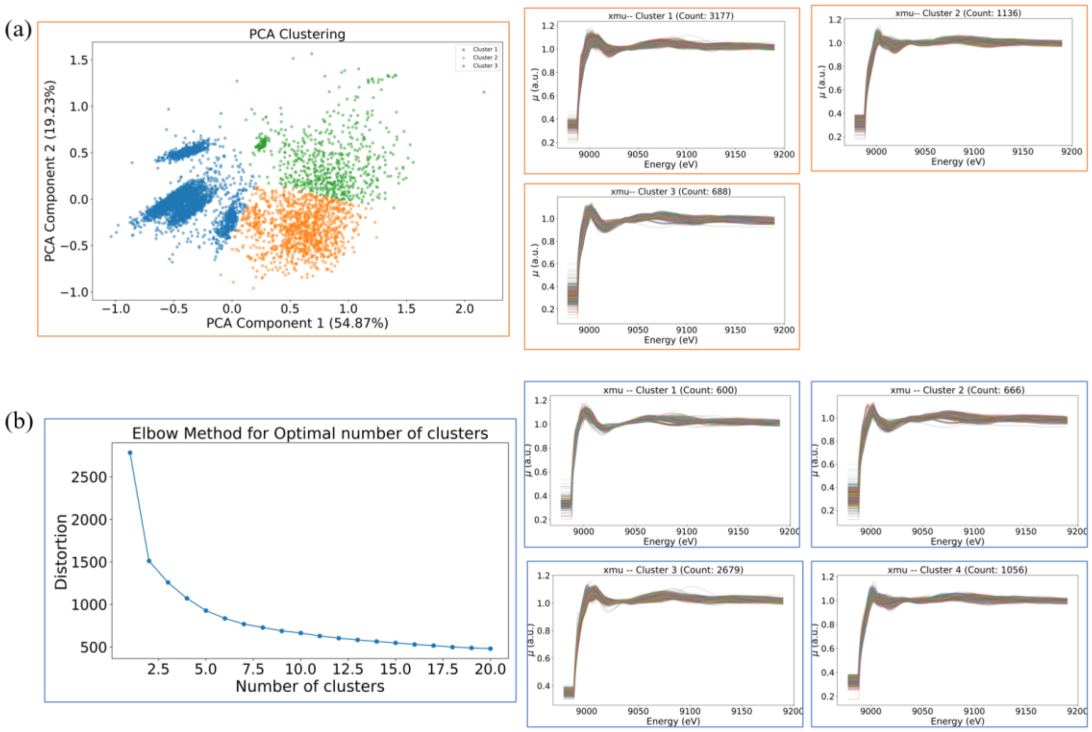

Figure S5 Clustering analysis of the Cu system before outlier removal. (a) Scatter plot of the first two principal components obtained from PCA applied to μ spectra, with data points grouped into three clusters using the K-means algorithm. (b) Identification of four K-means clusters for μ spectra using the elbow method.

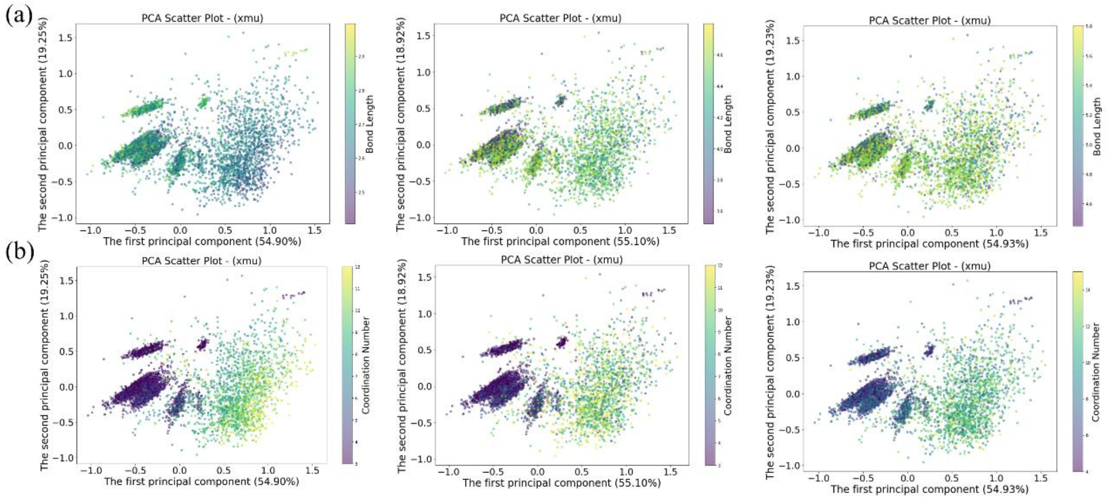

Figure S6 Principal-component analysis of the μ(E) spectra. (a) Score plot of the first two principal components colour-mapped by the first, second and third-shell average CR; CR, and (b) the corresponding plot colour-mapped by the first, second and third-shell average CN.

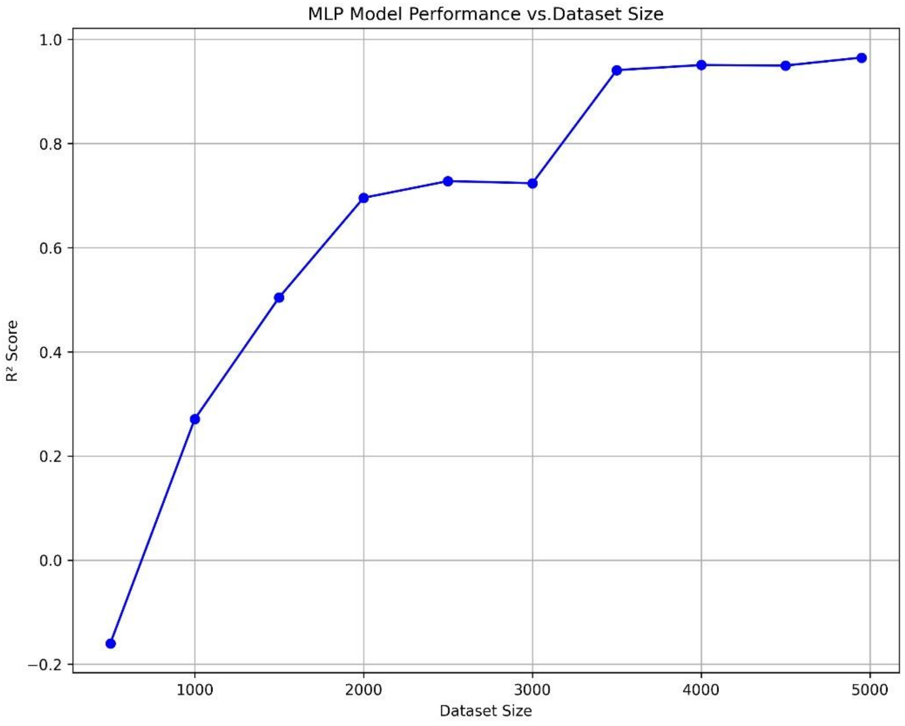

Figure S7 Evaluation metrics (R^2) for predicting CN in Cu systems using MLP as a function of dataset size.

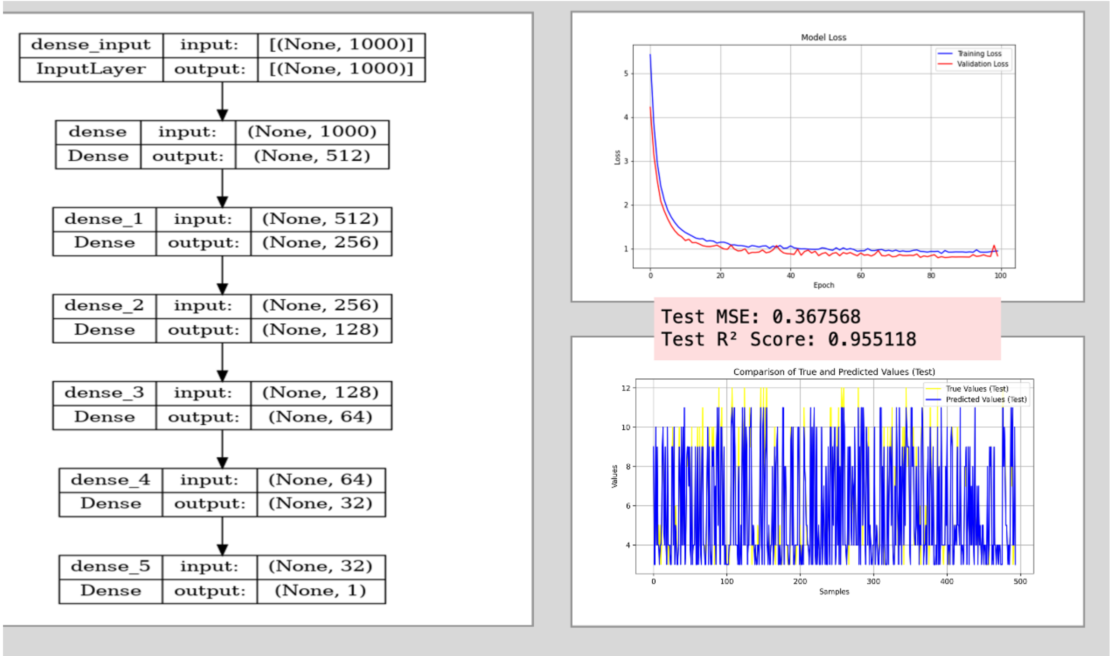

Figure S8 Prediction of the first shell CN in the Cu system using the MLP model, illustrates the prediction accuracy and the corresponding error metrics for the model's performance on the Cu dataset.

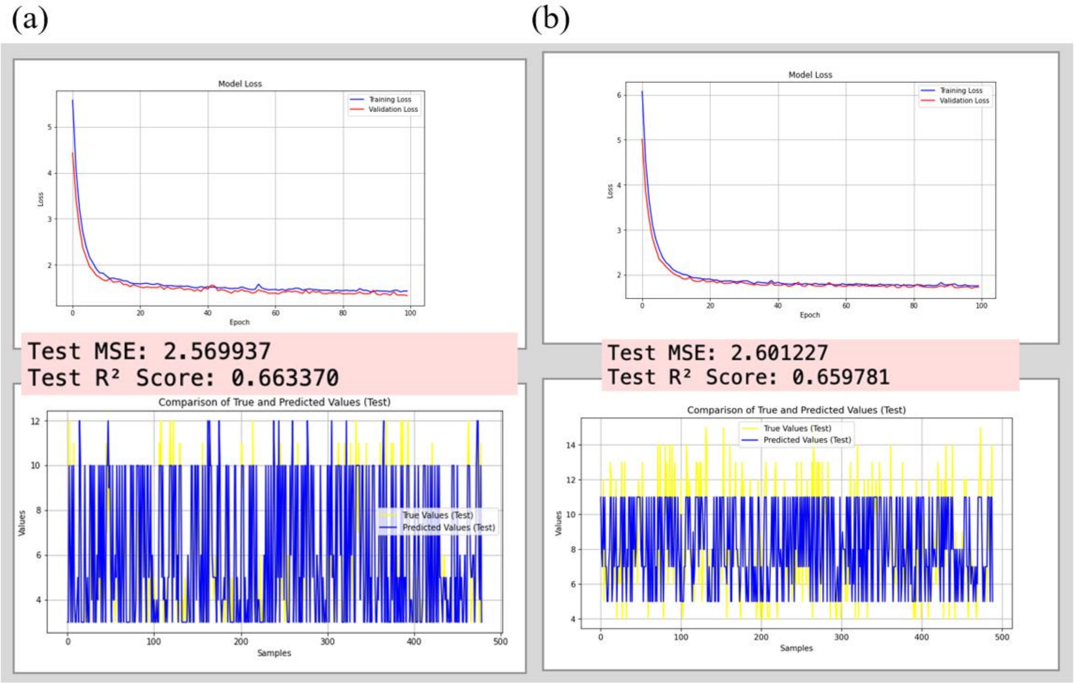

Figure S9 Prediction of CN in the Cu system using the MLP model, including the error metrics and visual comparison between predicted and true values. (a) Second-shell CN, (b) Third-shell CN.

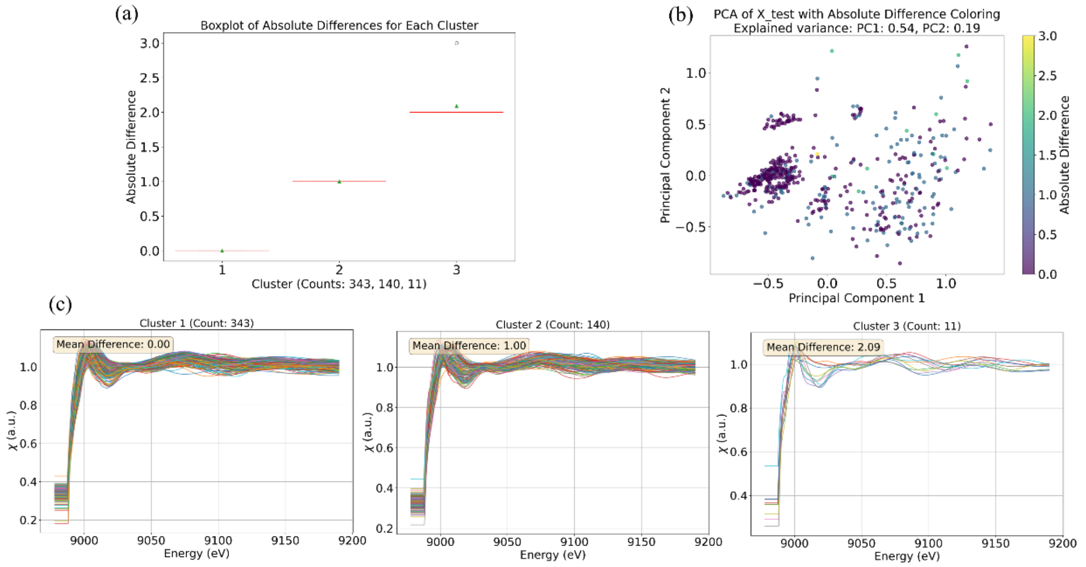

Figure S10 Prediction result analysis for the Cu system using XASDAML. (a) K-means clustering of prediction errors across different data points. (b) PCA analysis of μ spectra, with samples colored according to their prediction errors, providing an intuitive assessment of the model's overall performance. (c) μ spectra plots for each cluster obtained from the K-means clustering of prediction errors.

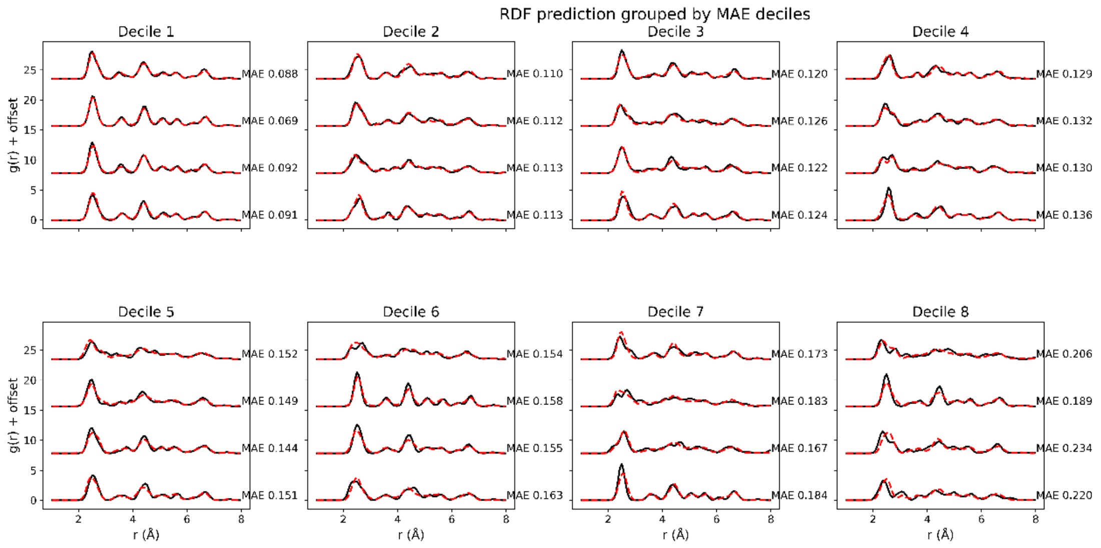

Figure S11 Performance of the random-forest model on the test set, binned by prediction error. The test spectra were ranked by their RDF MAE, split into eight equal-sized deciles, and four samples were randomly selected from each decile. In every subplot the black solid line is the reference RDF and the red dashed line is the model prediction; curves are vertically offset for clarity. The MAE for each sample is annotated on the right.

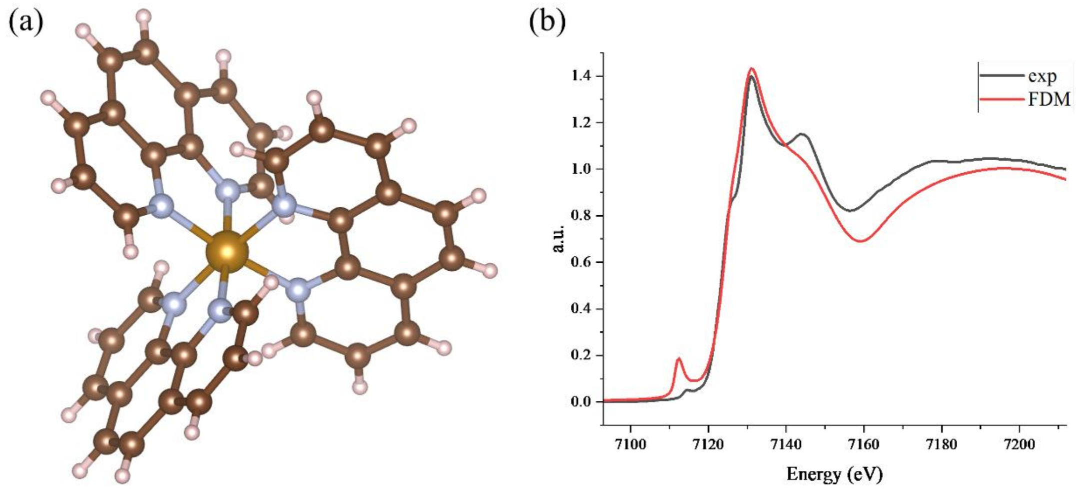

Figure S12 Reference structure and FDMNES validation. (a) Low-spin ground-state geometry of Fe II(phen)$_3$ taken from Yan et al. (2000) and used as the starting point for all FDMNES calculations. (b) Comparison of the calculated XANES for the structure in (a) (Γ_{hole} = 1.33 eV; red) with the experimental laser-off spectrum (black), demonstrating that the theoretical line shape reproduces the measured data.
